# Environmental Factors Influencing White-Tailed Deer (*Odocoileus virginianus*) Exposure to Livestock Pathogens in Wisconsin

**DOI:** 10.1371/journal.pone.0128827

**Published:** 2015-06-01

**Authors:** Shelli Dubay, Christopher Jacques, Nigel Golden, Bryant Kern, Kathleen Mahoney, Andrew Norton, Devi Patnayak, Timothy Van Deelen

**Affiliations:** 1 College of Natural Resources, University of Wisconsin-Stevens Point, Stevens Point, Wisconsin, United States of America; 2 Department of Biological Sciences, Western Illinois University, Macomb, Illinois, United States of America; 3 Florida Fish and Wildlife Conservation Commission, Holt, Florida, United States of America; 4 Department of Forest and Wildlife Ecology, University of Wisconsin-Madison, Madison, Wisconsin, United States of America; 5 Veterinary Diagnostic Laboratory, University of Minnesota, St. Paul, Minnesota, United States of America; Texas A&M Veterinary Medical DIagnostic Laboratory, UNITED STATES

## Abstract

White-tailed deer (*Odocoileus virginianus*) are commonly exposed to disease agents that affect livestock but environmental factors that predispose deer to exposure are unknown for many pathogens. We trapped deer during winter months on two study areas (Northern Forest and Eastern Farmland) in Wisconsin from 2010 to 2013. Deer were tested for exposure to six serovars of *Leptospira interrogans* (*grippotyphosa*, *icterohaemorrhagiae*, *canicola*, *bratislava*, *pomona*, and *hardjo*), bovine viral diarrhea virus (BVDV-1 and BVDV-2), infectious bovine rhinotracheitis virus (IBR), and parainfluenza 3 virus (PI3). We used logistic regression to model potential intrinsic (e.g., age, sex) and extrinsic (e.g., land type, study site, year, exposure to multiple pathogens) variables we considered biologically meaningful to exposure of deer to livestock pathogens. Deer sampled in 2010–2011 did not demonstrate exposure to BVDV, so we did not test for BVDV in subsequent years. Deer had evidence of exposure to PI3 (24.7%), IBR (7.9%), *Leptospira interrogans* serovar *pomona* (11.7%), *L*. *i*. *bratislava* (1.0%), *L*. *i*. *grippotyphosa* (2.5%) and *L*. *i*. *hardjo* (0.3%). Deer did not demonstrate exposure to *L*. *interrogans* serovars *canicola* and *icterohaemorrhagiae*. For PI3, we found that capture site and year influenced exposure. Fawns (n = 119) were not exposed to *L*. *i*. *pomona*, but land type was an important predictor of exposure to *L*. *i*. *pomona* for older deer. Our results serve as baseline exposure levels of Wisconsin white-tailed deer to livestock pathogens, and helped to identify important factors that explain deer exposure to livestock pathogens.

## Introduction

Serologic data are commonly used to infer disease status and infection history in free-ranging wildlife [1]. Because animals are actively shedding pathogens for short periods of time when compared to antibody persistence, biologists often look for evidence of previous exposure in hosts to investigate epizootiological questions [2]. In the midwestern United States, white-tailed deer (*Odocoileus virginianus*) are the most culturally and economically important game species [3, 4], and diseases that influence populations are of interest to state game agencies and hunters. White-tailed deer are commonly exposed to disease agents that affect both free-ranging wildlife and domestic animals. Antibody against several livestock pathogens, including *Leptospira* sp., infectious bovine rhinotracheitis, bovine viral diarrhea virus, and parainfluenza 3 virus have been previously identified in free-ranging white-tailed deer [5, 6].

*Leptospira interrogans* is a bacterial pathogen that is most commonly transmitted to wildlife and humans through contaminated drinking water [7]. Bacteria are heavily shed in urine that then contaminates the environment and can survive for months in moist environments [7]. *Leptospira* spp. affect a range of wild and domestic mammalian hosts, including deer. Infection can cause hemorrhage, jaundice, hepatitis, abortion or stillbirth, and death in cattle and deer [7, 8, 9]. Numerous serovars (differentiated by the presence of antigenic proteins on the outer sheath of the bacteria) of *L*. *interrogans* have been identified but serovars *grippotyphosa*, *icterohaemorrhagiae*, *canicola*, *bratislava*, *pomona*, and *hardjo* are associated with domestic and wild animals in the United States [7]. Wolf et al. (2008) found antibody against at least one serovar from as high as 23% of white-tailed deer sampled from two sites in southern Minnesota [10]. Also, seroprevalence of *L*. *i*. *pomona* in white-tailed deer has been positively related to seroprevalence in cattle sampled from the same county in Ohio [11].

Bovine viral diarrhea virus (BVDV) is a pestivirus that can cause severe diarrhea, abortion, stillbirth, and death in cattle [12, 13]. Two serotypes are described, BVDV type 1 (BVDV-1) and type 2 (BVDV-2). Van Campen et al. (2001) stated that a population size large enough to provide a consistent source of infection is likely necessary for BVDV to circulate in free-ranging ungulates but little is known about the epidemiology of BVDV in white-tailed deer [13]. Wolf et al. (2008) identified antibody against BVDV-1 in 46% and 31% of white-tailed deer sampled in two study areas in Minnesota and identified BVDV-2 antibodies in 28% and 7% of deer sampled in these areas [10]. Additionally, Chase et al. (2008) identified clinical BVDV infection in two white-tailed deer in South Dakota [14].

Infectious bovine rhinotracheitis virus (IBR) is a herpesvirus that causes upper respiratory infection with viral pneumonia, but also has been associated with abortions and ocular impairments in cattle [15]. The virus is most commonly identified in cattle herds in feedlots, so density may play a role in epidemiology of the disease in cattle [15]. Higher density white-tailed deer populations on cattle ranches in Mexico had higher seroprevalence against IBR [5]. Ingebrigtsen et al. (1986) identified IBR antibody in 15% of white-tailed deer tested in Minnesota, and adult deer had a significantly greater seroprevalence than fawns [6]. Lamontagne et al. (1989) identified 53% seroprevalence in white-tailed deer on Anticosti Island, Quebec and older animals were more often exposed than younger individuals (<1.5 years old) [16]. Additionally, high mortality of white-tailed deer on the island was attributed to an epizootic of IBR, though low food availability and harsh winters may have decreased the immune competency of deer and therefore contributed to the epidemic [17].

Parainfluenza 3 virus (PI3) is classified as a single-stranded pleomorphic RNA virus of the Family Paramyxovirus [18]. The clinical signs of PI3 in cattle include fever, cough, watery nasal and lacrimal discharge, as well as an increased respiration rate [19]. Infection can increase morbidity of other viral diseases such as bovine viral diarrhea and infectious bovine rhinotracheitis. The impact of infection is more significant when coupled with secondary bacterial pneumonia [18, 19]. Parainfluenza 3 virus is transmitted through aerosolization and contact with nasal fluids [20]. Little is known about the disease in deer, but it is thought to spread through contact with domesticated cattle. Previous serologic surveys have shown a high seroprevalence against PI3 in many wild ruminant populations, including white-tailed deer [6, 19, 21, 22].

The primary objectives of this study were to 1) determine if Wisconsin white-tailed deer are currently exposed to livestock pathogens and 2) identify environmental variables that influence exposure of white-tailed deer to livestock pathogens in Wisconsin. To our knowledge, serologic surveys for exposure of deer to livestock pathogens have not been conducted in Wisconsin since the 1970’s. Additionally, we aimed to identify if deer were commonly exposed to multiple livestock pathogens. Given similar environmental variables in study areas and behavioral characteristics of deer, we expected that some deer would show evidence of exposure to numerous livestock-associated pathogens. To our knowledge, our study is the first to include exposure to pathogens as explanatory variables in models that explain exposure of white-tailed deer to other livestock pathogens. Lastly, we identified environmental characteristics associated with deer exposed to livestock pathogens because how deer contribute to the transmission cycle of many livestock pathogens is largely unknown [5, 14].

## Materials and Methods

This research was conducted in strict accordance to the 2011 Guidelines of the American Society of Mammalogists for the use of Wild Mammals in Research. The Institutional Animal Care and Use Committee, Research Animal Resources Center, at the University of Wisconsin-Madison (Protocol Number: A01446-0-08-10) specifically approved this study, including all sampling methods. White-tailed deer that were mortally injured during capture were euthanized by gunshot to the head (approved in 2013 American Veterinary Medical Association euthanasia guidelines).

**Fig 1:**
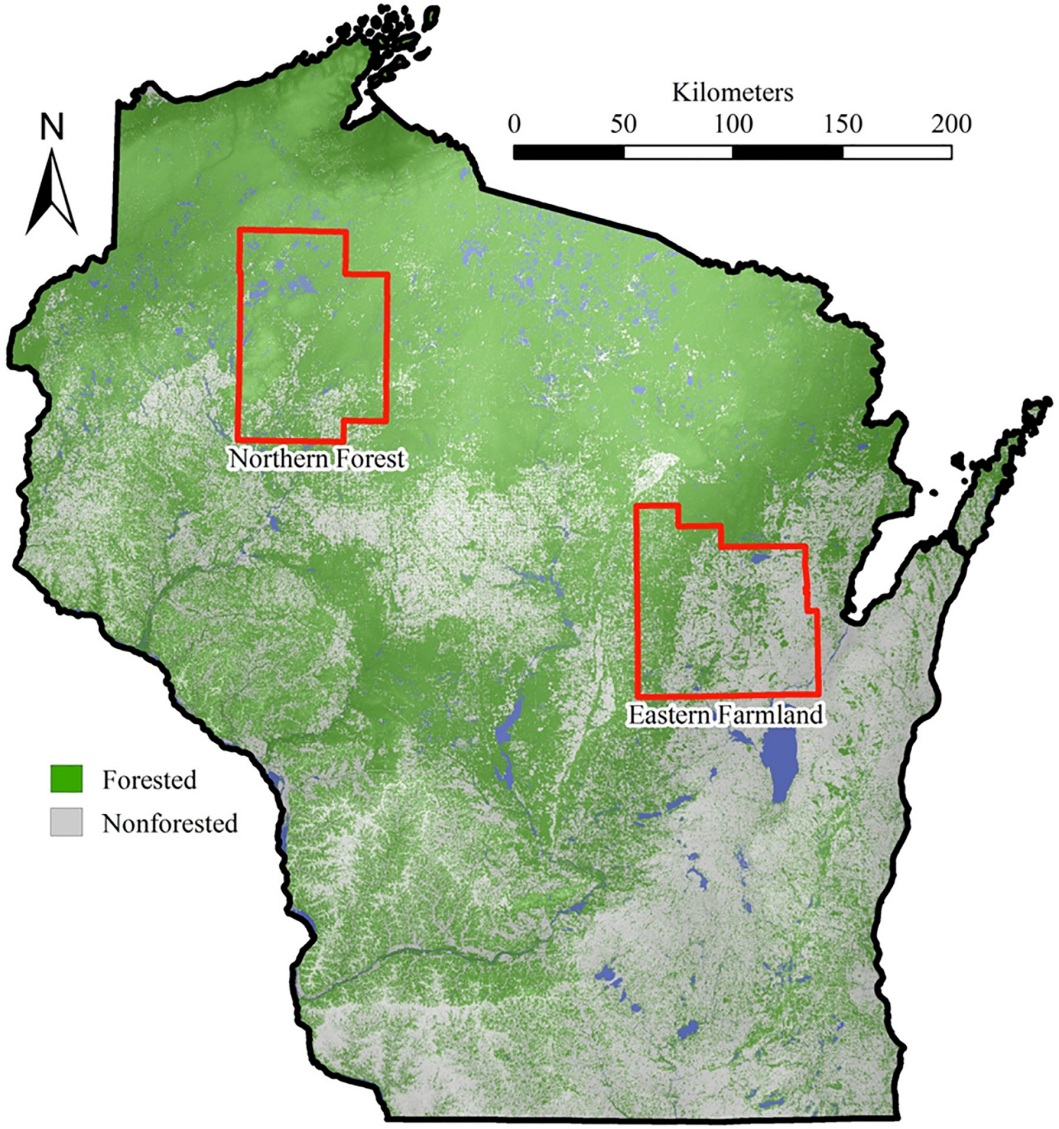
White-tailed deer (*Odocoileus virginianus*) sites of capture throughout northern and eastcentral Wisconsin, USA, 2010–2013. The thick black line delineates the state boundaries and thick red lines delineate study area (e.g., northern forest, eastern farmland) boundaries (Created by A. Norton using ArcMap 9.2. Forestland: Wisconsin Department of Natural resources 1998 WISCLAND land cover, Madison WI. County Outlines: U.S. Census Bureau. 1999 Wisconsin County Outlines, Madison, WI).

White-tailed deer were trapped concurrently in two study areas in Wisconsin selected to represent regions where habitat type and deer densities differed (Fig 1). We trapped deer on private land, and the owner of each parcel granted us permission to trap. The Wisconsin Department of Natural Resources granted permission to trap deer on state lands and the United States Forest Service granted permission for trapping on the Chequamegon-Nicolet National Forest. Deer trapping on Wisconsin County Forest lands was approved by the Wisconsin County Forest Association. Deer trapping on county land was approved by Rusk County and Sawyer County. We did not trap on tribal lands and endangered species were not captured.

The Northern Forest study area (5,905 km^2^) included Sawyer and Rusk counties, and 29% was publicly owned (e.g., county, state, or federal) and 5% was under tribal ownership. An additional 10% of the study area was private land enrolled in Open Managed Forest Law, so the public had access for hunting. Much of the landscape (78%) consisted of forested or shrubland habitat, including northern hardwoods (*Acer* spp., *Betula* spp., *Tilia americana*, *Quercus* spp., *Fagus americana*, *Tsuga canadensis*, *Pinus* spp., *Abies balsamia*, *Populus* spp., *Picea glauca*), tamarack-black spruce (*Larix laricina*, *Picea mariana*) bogs, northern white cedar (*Thuja occidentalis*) swamps, and alder (*Alnus* spp.) swamps. Primary land use in the area was forestry, and topography was moderately rolling hills [23]. Soils included stony glacial till, pitted outwash sands, and peat. Deer habitat quality has declined since the 1930s with the maturation of forests. Deer population goals in the Northern Forest currently average approximately 70% of carrying capacity [23].

The Eastern Farmland study area (6,003 km^2^) included Shawano, Waupaca, and Outagamie counties, and 2% of the land area was publicly owned, and 0.7% was private land enrolled in Open Managed Forest Law. Thirty-five percent of the landscape was forested, including floodplain forests and lowland hardwood swamps (*Acer* spp., *Quercus* spp., *Ulmus rubra*, *Fraxinus* spp. *Tilia americana*, *Salix* spp.), and cattail (*Typha* spp.) or sedge (*Carex* spp.) wetlands. Primary land use throughout the area was row crop agriculture, and topography was dominated by gently rolling hills [23]. Deer population goals in the Eastern Farmland have increased substantially since the early 1960s and current goals average 20–40% of estimated carrying capacity [23].

We captured deer over three sampling years between December and April, 2010–2013 using Stephenson box traps [24], Clover netted-cage traps [25], modified rocket nets (Kiwi Captures International, Waukesha, WI; US Patent No. 7,398,617 B2), modified drop nets [26], dart guns, and helicopter net-gunning [27, 28]. Physically captured deer were either restrained and sampled, or immobilized by hand injection with 1.0–1.3 mg/kg xylazine hydrochloride and 3.0–4.0 mg/kg ketamine, intramuscular (IM) or intravenous (IV; jugular vein). All remotely darted deer were administered (IM) 2.4 mg/kg xylazine hydrochloride and 4.6 mg/kg Telazol (tiletamine hydrochloride and zolazepam hydrochloride) via pressurized darts projected using CO_2_ powered injection rifles. Prior to release, all chemically immobilized deer were administered (IV or IM) an antagonist to the xylazine consisting of 2 mg/kg tolazoline hydrochloride [29].

We collected blood samples from deer via jugular venipuncture and separated sera from cells in the field via centrifugation [5]. For recaptured deer, we took blood only upon their initial capture. Sera were sent to the Minnesota Veterinary Diagnostic Laboratory and were tested for antibody against six serovars (*grippotyphosa*, *icterohaemorrhagiae*, *canicola*, *bratislava*, *pomona*, and *hardjo*) of *Leptospira interrogans* using microscopic agglutination, against BVDV and IBR using serum neutralization, and against PI3 using hemagglutination inhibition [7, 11, 30]. These tests use either bacterial antigen or respective virus which will react with antibodies if present. The initial dilution of sera for serum neutralization tests for IBR and BVDV antibody was 1:8 and sera were diluted two-fold until 1:8,192. For BVD virus, Singer strain (genotype 1) and strain 125 (genotype 2) were used and for IBR strain Colorado was used. The initial dilution of sera for the hemagglutination inhibition test for PI3 antibody was 1:10 and sera were diluted two-fold until 1:640. The Reisinger SF-4 strain was used for the test. Viruses were obtained from the National Veterinary Services Laboratory. An antibody titer ≥ 1:100 was considered positive for exposure to *Leptospira interrogans*, a titer of ≤ 1:10 was considered positive for exposure to PI3, and titers ≥ 1:8 were considered evidence of exposure to IBR and BVDV [11, 16, 17, 31].

### Analyses

An information-theoretic modeling approach was used to identify relationships between variables and white-tailed deer exposure to livestock pathogens. We conducted logistic regression analyses on exposure to PI3 and *L*. *i*. *pomona* (S1 File). We posited biologically plausible intrinsic (e.g., age, sex) and extrinsic (e.g., land type, study site, year, evidence of exposure to multiple infectious diseases) variables we considered influential in deer exposure to infectious diseases (Table 1). Prior to modeling, we screened all predictor variables for collinearity using Pearson’s correlation coefficients (*r* > |0.5|) and used quantile plots to evaluate assumptions of normality; we used one variable from a set of collinear variables for modeling [32]. For PI3 and *L*. *i*. *pomona*, we specified 15 models: a global model containing 7 variables and a subset of *a priori* models. We also developed models that included the interaction of variables (e.g., site, year, land type) to account for multiple factors potentially influencing disease exposure simultaneously. Logistic regressions were used to analyze models that represented competing hypotheses explaining exposure to PI3 or *L*. *i*. *pomona*. However, all possible combinations of variables were not considered as this inflates the number of models beyond what can be reliably analyzed [33].

**Table 1:** Variables used for modeling exposure of white-tailed deer (*Odocoileus virginianus*) to livestock pathogens within two sites in Wisconsin, USA, 2010–2013.

Variable	Description
**Land type (LT)**	Trap location on public or private land
**Age**	Deer age (Fawn, yearling, 2.5 or older)
**Capture Site (CS)**	Eastern Farmland or Northern Forest
**Capture Year (CY)**	2011, 2012, or 2013
**Sex**	Male or female deer
**IBR**	Exposure to infectious bovine rhinotracheitis virus
**PI3**	Exposure to parainfluenza 3 virus
**LP**	Exposure to *L*. *i*. *pomona*

We used Akaike’s Information Criterion (AIC) to select models that best described the data and used Akaike weights (*w*_*i*_) as a measure of relative support for each model [33]. Because the number of deer tested was small relative to the number of variables (*K*) in several models (i.e., *n/K* < 40), we used AIC corrected for small sample size (AIC_*c*_) for model selection [33]. We used formulas presented in Burnham and Anderson (2002) to calculate AIC_*c*_ from the log-likelihoods for each model [32]. We compared AIC_*c*_ values to select the most parsimonious model and considered models differing by ≤2 ΔAIC_*c*_ from the selected model as competitive models [33, 34]. We examined models ≤2 ΔAIC_*c*_ from the best model to ascertain if they differed by 1 parameter from the best model and had essentially the same maximized loglikelihood [34]. In this case, models with additional parameters were unsupported and noncompetitive and considered potential models only because they contributed an additional parameter [33, 34]. To account for model selection uncertainty among competitive models, we used model averaging to generate unconditional parameter estimates; parameter estimates were unconditional in the sense that they were conditioned on a suite of models weighted by their respective AIC_c_ scores rather than on a single model whose probability of being the highest-ranked model may be only marginally higher than next-ranked models [32, 33].

We determined associations between response and predictor variables using odds ratios (OR); OR for predictor variables are the relative amount by which the odds of the outcome increase (OR>1.0) or decrease (OR<1.0) with each unit increase in the predictor variable [35].

Further, we determined predictive capabilities of models with area under the receiver operating characteristic (ROC) curve. We considered ROC values ≥ 0.8 excellent discrimination, 0.7–0.8 acceptable discrimination, 0.5–0.7 as low discrimination, and values ≤0.5 indicated that model predictive capabilities were no better than random [36, 37, 38].

## Results

During the 2010–2011 trapping season, deer (n = 133) did not show evidence of exposure to BVDV-1 or BVDV-2. As a result, we did not test for exposure to BVDV in subsequent years. Most animals had relatively low titers to the pathogens tested, but several deer had higher titers to PI3. For PI3, 14 deer had titers of 1:10, 33 deer had titers of 1:20, 8 deer had titers of 1:40, 10 deer each had titers of 1:80 and 1:160, and 3 deer had titers of 1:320. *For L*. *i*. *pomona*, 13 deer had titers of 1:100, 17 deer had titers of 1:200, 5 deer had titers of 1:400 and 2 deer had titers of 1:800.

Table 2 shows seroprevalence of PI3, *L*. *i*. *pomona*, and IBR with reference to variables tested in Table 1 (S1 File). We tested 315 deer sera for exposure to IBR and 25 (7.9%) were exposed. We tested 316 sera for exposure to *Leptospira interrogans* serovar *pomona* and 37 (11.7%) showed evidence of exposure. Only 3 of 315 (1.0%) deer had evidence of exposure to *L*. *i*. *bratislava*, 8 of 316 (2.5%) had evidence of exposure to *L*. *i*. *grippotyphosa*, and 1 of 316 (0.3%) had evidence of exposure to *L*. *i*. *hardjo*. Deer did not demonstrate exposure to *L*. *interrogans* serovars *canicola* and *icterohaemorrhagiae*. We tested 316 sera for exposure to PI3 and 78 (24.7%) showed evidence of exposure.

**Table 2:** Seroprevalence (%) of white-tailed deer for PI3, *L*. *i*. *pomona*, and IBR from 2010–2013 in 2 areas in Wisconsin in comparison to environmental variables.

Variable	PI3	*L*.*i*. *pomona*	*IBR*
**Overall** ^**a**^	24.7 (78/316)	11.7 (37/316)	7.9 (25/315)
**Northern Forest**			
Year 1	35.9 (23/64)	25.0 (16/64)	12.5 (8/64)
Year 2	2.1 (1/48)	10.4 (5/48)	12.5 (6/48)
Year 3	6.5 (2/31)	12.9 (4/31)	3.2 (1/31)
**Eastern Farmland**			
Year 1	25.7 (18/70)	7.1 (5/70)	2.8 (2/69)
Year 2	25.0 (14/56)	5.3 (3/56)	12.5 (7/56)
Year 3	42.6 (20/47)	8.5 (4/47)	2.1 (1/47)
**Age**			
0.5 years	20.2 (24/119)	0.0 (0/119)^b^	3.4 (4/119)
1.5 years	23.6 (17/72)	18.1 (13/72)	9.7 (7/72)
2.5 + years	29.6 (37/125)	17.6 (22/125)	11.3 (14/124)
**Sex**			
Male	24.8 (35/141)	6.4 (9/141)	5.6 (8/141)
Female	24.6 (43/175)	16.0 (28/175)	9.8 (17/174)
**Land Type**			
Public	17.8 (18/101)	21.8 (22/101)	13.0 (13/100)
Private	27.9 (60/215)	7.0 (15/215)	5.6 (12/215)
**PI3**			
Exposed	N/A	12.8 (10/78)	9.0 (7/78)
Unexposed	N/A	11.3 (27/238)	7.6 (18/237)
***L*. *i*. *pomona***			
Exposed	27.0 (10/37)	N/A	13.9 (5/36)
Unexposed	24.3 (68/279)	N/A	7.2 (20/279)
**IBR**			
Exposed	28.0 (7/25)	20.0 (5/25)	N/A
Unexposed	24.5 (71/290)	10.7 (31/290)	N/A

^a^Sample sizes vary because quantity of serum did not allow for all tests for some deer.

^b^Fawns omitted from logistic regression analyses.

Logistic regression models explaining exposure of white-tailed deer to PI3 are listed in Table 3 in order of their ranking. Of 15 logistic regression models explaining exposure to PI3, a model containing CS, CY, and the interaction between CS and CY was selected as the highest ranked model and parameter estimates (β) for CS 1, and the CS 1 * CY 1 did not have 95% confidence intervals that included zero (Table 4). Support for this model was substantial (w_i_ = 0.998) and predictive capability of the model was acceptable (ROC = 0.70); all other models were noncompetitive (ΔAIC_*c*_ ≥ 12.49, *w*_*i*_ < 0.02). The logistic equation for this model was logit(μ) = –0.300–2.374 (CS 1)– 0.761 (CY 1)– 0.799 (CY 2) site) + 2.857 (CS 1 * CY 1)– 0.377 (CS 1 * CY 2; Table 5).

**Table 3:** Logistic regression models explaining exposure of 315 white-tailed deer (*Odocoileus virginianus*) to parainfluenza 3 virus within two sites in Wisconsin, USA, from 2010–2013.

Model^a^	AIC_*c*_ ^b^	ΔAIC_*c*_ ^c^	*w*_*i*_ ^d^	*K* ^e^	ROC^f^
CS, CY, CS*CY	327.32	0.00	0.998	6	0.70
CS, CY, LT, CS*CY*LT	339.81	12.49	0.002	7	0.66
**CS, CY**	345.60	18.28	0.00	4	0.63
CS, LT, CS*LT	349.78	22.47	0.00	4	0.62
**CY**	349.74	22.42	0.00	3	0.60
**Global (Age, Sex, CS, LT, CY, LP, IBR)**	351.16	23.84	0.00	10	0.67
**CS**	351.17	23.86	0.00	2	0.58
**LP, CY**	351.79	24.47	0.00	4	0.60
**CS, IBR**	352.12	24.80	0.00	3	0.59
**CS, LP**	352.60	25.29	0.00	3	0.59
**LT**	353.30	25.99	0.00	2	0.56
**IBR, LP, CS**	353.42	26.10	0.00	4	0.60
**CS, LP, LT**	353.55	26.24	0.00	4	0.60
**IBR, LP**	358.37	31.06	0.00	3	0.52
Sex, Age, Sex*Age	360.79	33.48	0.00	6	0.57

^a^CS = capture site; CY = capture year; LT = land type (public vs. private); Age = deer age; Sex = male or female; LP = Exposure to *L*. *i*. *pomona;* IBR = exposure to infectious bovine rhinotracheitis virus.

^b^ Akaike’s Information Criterion corrected for small sample size [33].

^c^ Difference in AIC_*c*_ relative to minimum AIC.

^d^ Akaike weight [33].

^e^ Number of parameters.

^f^ Receiver Operating Curve.

**Table 4:** Logistic regression models explaining exposure of 196 white-tailed deer (*Odocoileus virginianus*) to *Leptospira interrogans pomona* from two sites in Wisconsin, USA, 2010–2013.

Model^a^	AIC_*c*_ ^b^	ΔAIC_*c*_ ^c^	*w*_*i*_ ^d^	*K* ^e^	ROC^f^
**LT**	184.89	0.00	0.278	2	0.64
CS, LT, CS*LT	185.61	0.72	0.194	4	0.66
**IBR, PI3, LT**	186.44	1.55	0.128	4	0.63
**LT, Age**	186.71	1.82	0.112	3	0.66
LT, CY, LT*CY	187.09	2.20	0.093	6	0.70
**CS, Age, LT**	187.10	2.21	0.092	4	0.67
**CS**	188.49	3.60	0.046	2	0.61
**CS, Age**	190.46	5.57	0.017	3	0.62
**CS, CY**	191.37	6.48	0.011	4	0.63
**CS, Age, Sex**	192.20	7.31	0.007	4	0.61
**Global (Age, Sex, CS, LT, CY, PI3, IBR)**	192.61	7.72	0.006	9	0.69
**Sex**	192.77	7.87	0.005	2	0.55
Site, Age, Sex, Site*Age*Sex	193.17	8.28	0.004	5	0.62
**PI3**	194.38	9.49	0.002	2	0.50
PI3, IBR, PI3*IBR	194.65	9.76	0.002	4	0.53

^a^LT = land type (public vs. private); CS = capture site; IBR = exposure to infectious bovine rhinotracheitis virus; PI3 = exposure to parainfluenza 3 virus; Age = deer age; Year = capture year; Sex = male or female.

^b^Akaike’s Information Criterion corrected for small sample size [31].

^c^Difference in AIC_*c*_ relative to minimum AIC.

^d^Akaike weight [31].

^e^Number of parameters.

^f^Area under the receiver operating characteristic curve.

**Table 5:** Parameter estimates (β), standard error (SE), odds ratio, and odds ratio 95% confidence intervals (lower CI and upper CI) for the best-approximating models in candidate sets evaluated for exposure of white-tailed deer to PI3 and *L*. *i*. *pomona* in northern and east-central Wisconsin, USA, 2010–2013.

Parameter^a^	β	SE	Odds ratio^b^	Lower CI	Upper CI
**PI3**					
Intercept	–0.300	0.295			
CS 1	–2.374	0.788	0.093	0.020	0.437
CY 1	–0.761	0.402	0.467	0.212	1.028
CY 2	–0.799	0.427	0.450	0.195	1.039
CS1 × CY1	2.857	0.874	17.406	3.138	96.562
CS1 × CY2	–0.377	1.318	0.686	0.052	9.083
**LP** ^c^					
Intercept	1.954	0.048			
LT	–1.002	0.035	0.367	0.105	0.629

^a^CS 1 = Northern Forest study site 1, CS 2 = Eastern Farmland study site 2, CY 1 = 2011, CY 2 = 2012.

^b^Odds ratios used to estimate measures of association between variables. A measure of association in which a value near 1 indicates no relationship between variables [36].

^c^Model-averaged parameter, SE, odds ratio, and odds ratio 95% confidence intervals.

Fawns (n = 119) did not demonstrate evidence of exposure to *L*. *i*. *pomona*, thus we omitted this cohort from our analyses. Logistic regression models explaining exposure of white-tailed deer to *L*. *i*. *pomona* are listed in Table 4 in order of their ranking Of 15 logistic regression models explaining exposure to *L*. *i*. *pomona*, a model containing land type was the highest-ranked model, though support for this model was not substantial (w_i_ = 0.278) and predictive capability of the model was low (ROC = 0.64). Several other models were < 4.0 ΔAIC units from the highest-ranked model, thus we generated parameter estimates across competitive models. Model-averaged parameters estimated for the top model suggested that exposure to *L*. *i*. *pomona* was influenced by land type (β = –1.002, 95% confidence interval = –1.971 to—0.034). However, parameter estimates for the remaining covariates (IBR, PI3, CS, age, CY, sex) were inconsistent, 95% confidence intervals always overlapped zero, and P-values based on permutation tests for these covariates were not significant (P ≥ 0.07), suggesting these factors had little effect on exposure of white-tailed deer to *L*. *i*. *pomona*. The logistic equation for the highest-ranked model was logit(μ) = 1.954–1.002 (LT; Table 5).

Probability of exposure to parainfluenza 3 virus was 0.917 (OR = 0.093, 95% CI = 0.020–0.437) lower in the Northern Forest than the Eastern Farmland study site (Table 5). Conversely, probability of exposure to parainfluenza 3 was 17.41 (OR = 17.406, 95% CI = 3.138–96.562) times greater in the Northern Forest than the Eastern Farmland study site during 2011 (Table 5). For the remaining categorical variables in the highest-ranked parainfluenza 3 exposure model, odds ratio 95% confidence intervals overlapped each other, indicating that these were poor predictors of exposure to parainfluenza 3 (Table 5). Moreover, probability of exposure to *L*. *i*. *pomona* decreased by 0.37 (OR = 0.367, 95% CI = 0.105–0.629) between private and public land (Table 5).

## Discussion

In the white-tailed deer sampled, we identified antibodies against several pathogens that can affect livestock and white-tailed deer, and in some cases, humans [7, 8, 17, 19]. Serologic evidence of exposure to PI3 and *L*. *i*. *pomona* occurred in over 10% of deer sampled, but contrary to Wolf et al. (2008), who sampled deer in adjacent Minnesota, we did not identify BVDV antibody in deer captured in two areas in Wisconsin [10]. Wolf et al. (2008) sampled 114 deer and found 20–39% seroprevalence, thus it is reasonable to assume that, if present, we would have detected antibodies given our sampling intensity (n = 133 deer) [10]. Similar serologic investigations have found low seroprevalence in white-tailed deer. Passler et al. (2008) identified antibody in 2 of 165 (1.2%) white-tailed deer in Alabama [39]. Additionally, Sadi et al. (1991) did not identify antibody in 396 deer sera from Anticosti Island, Quebec and speculated that the deer had not been exposed because domestic ruminants had not been on the island for over 50 years [17]. Cantu et al. (2008) tested 331 deer sera in 2004 for antibodies against BVDV from 15 different cattle ranches in Mexico and found that seroprevalence varied from 11.1% to 100% depending upon the site of capture [5]. Myers et al. (2015) sampled mule deer (*O*. *hemionus*) sera in Washington for BVDV antibody and found similar seroprevalence in 2000 (53%) and 2001 (59%), suggesting little variability with year [40]. White-tailed deer may be exposed only sporadically to BVDV depending upon site and/or year and we may not have sampled during a year with obvious exposure. Cattle have been shown to experimentally transmit BVDV to white-tailed deer in close proximity, and fawns born to adults exposed to infected cattle were persistently infected with BVDV [41], so perhaps deer are not coming in contact with infected cattle on our study sites.

White-tailed deer are commonly exposed to PI3, and we found that 25% of the deer sera from Wisconsin had antibodies against PI3, similar to that reported previously for mule deer trapped on national parks (32%) [42]. In Minnesota, Ingebrigtsen et al. (1986) found that 20% of the white-tailed deer tested were seropositive for PI3 [6]. Seroprevalences documented in this study and from Minnesota are lower than what has been reported in other areas. Seroprevalence of 82 to 84% has been reported for white-tailed deer in Quebec in the 1980’s [17], and seroprevalence of 95% was reported for mule deer in Idaho in 1975 [43]. Exposure to PI3 may depend upon local environmental factors, including deer and/or cattle density.

Deer in the Eastern Farmland study site were more commonly exposed to PI3 (30%) than those captured in the Northern Forest (18%), and both the number of cattle and the number of deer are higher in the eastern study area. In December 2012, over 103,000 cattle were registered with the National Agriculture Statistics Service (NASS; [44]) from Waupaca, Outagamie, and Shawano counties, which includes the Eastern Farmland study area (Fig 1). However, approximately 18,000 cattle were registered with NASS in December 2012 in Rusk and Sawyer counties where Northern Forest deer were captured [44]. Parainfluenza 3 is likely transmitted to deer from cattle, however the disease also could be maintained in deer with no contact with cattle [18, 42]. A previous study has shown higher seroprevalence for livestock diseases in deer herds with higher densities [5]. In 2012, fall deer densities for the three deer management units closest to Shiocton, Wisconsin (e.g., where Eastern Farmland deer were trapped) were 267, 197, and 194 deer per square km [45]. Fall deer densities in 2012 in deer management units near Winter, Wisconsin (e.g., where Northern Forest deer were trapped) were 73, 73, and 47 deer per square km [45]. Though speculative, increased seroprevalence of PI3 in the Eastern Farmland could be attributed to higher deer and/or cattle density, suggesting that host density affects exposure to livestock pathogens.

Our findings indicate that seroprevalence for PI3 varies with year. Previous studies have documented significant variation in PI3 seroprevalence in free-ranging ungulates by year [46]. We found similar seroprevalence in 2011 (41/134 = 31%) and 2013 (22/78 = 28%), but seroprevalence was lower in 2012 (15/104 = 14%), but prevalence changed dramatically with year in the Northern Forest site. In 2011, prevalence was 36%, but dropped to 2% and 7% in 2012 and 2013, respectively, so the interaction between year and site was significant, indicating that seroprevalence varies independently on each site on a yearly basis. Myers et al. (2015) also found a difference in seroprevalence for PI3 with study site in the same year for mule deer [40]. Since both deer and cattle have been shown to transmit PI3 [20], perhaps deer and/or cattle are maintaining PI3 in the populations separately between the two study sites. Additional research is needed to better understand how cattle and/or deer density affect seroprevalence of PI3 in deer.

We found that 12% of the white-tailed deer sampled in Wisconsin had antibody against *L*. *i*. *pomona*. In Washington, Myers et al. (2015) found antibody against *Leptospira interrogans* serovars *grippotyphosa* (13%), *bratislava* (4%), and *canicola* (1%), but not serovar *pomona* [40]. However, Roug et al. (2012) found that 6.5% of the mule deer sera tested were seropositive for *Leptospira* antibody (all serovars combined) [47]. In Minnesota, only 3% of the white-tailed deer sera tested in 1976 were seropositive for all serovars of *Leptospira interrogans* combined [6]. From 1984 through 1989, Goyal et al. (1992) tested white-tailed deer sera for antibody against 6 serovars of *L*. *interrogans* in Minnesota [48]. Deer sera were not seropositive from 1984–1985, but all sera collected from 1986 through 1988 were positive for antibody against *L*. *i*. *pomona* and/or *L*. *i*. *bratislava*, suggesting variability in exposure by year [48]. Fournier et al. (1986) found that 7% of the white-tailed deer sera tested in Ohio had antibody against at least one serovar of *Leptospira*, and found that a higher proportion of cattle were seropositive from counties where seropositive deer were identified than from counties where deer were seronegative [11].

In our study, exposure of white-tailed deer to *L*. *i*. *pomona* was positively associated with public land. *Leptospira interrogans* serovar *pomona* is maintained in deer and other maintenance hosts, including wildlife and domestic animals. Myers et al. (2015) suggested that mule deer may be exposed to *L*. *interrogans* from contact with cattle that commonly share their range [40]. They also propose that mule deer could serve as maintenance hosts for the bacterium [40]. Cirone et al. (1978) found that over 75% of raccoons (*Procyon lotor*), coyotes (*Canis latrans*), and striped skunks (*Mephitis mephitis*) tested in California were seropositive for *L*. *i*. *pomona* antibody [49]. In the same study, Cirone et al. (1978) suggested that high seroprevalence in white-tailed deer was related to the site where deer were sampled which varied with access to contaminated water [49]. All black rats (*Rattus rattus*) sampled from a ring-necked pheasant (*Phasianus colchicus*) farm with access to fresh tapwater were seronegative, whereas Norway rats (*R*. *norvegicus*) sampled from sites with access to only stagnant water were seropositive for leprospiral antibodies [49]. White-tailed deer are positively associated with water, especially when moisture content of foods is low [50]. Additionally, home ranges for white-tailed deer in the midwestern United States often contain ponds, lakes, or other waterways [51]. At this time, we do not know how deer are exposed to *L*. *i*. *pomona*, but contaminated water sources on public land may play a role, specifically if maintenance hosts and white-tailed deer occur at higher densities at these water sources, and deer density has been proposed as a factor influencing seroprevalence of *L*. *i*. *pomona* [11].

We did not find evidence that individual white-tailed deer were exposed to numerous livestock pathogens during the same year and environmental variables that explained exposure to PI3 and *L*. *i*. *pomona* differed within our study. *Leptospira* sp. is transmitted primarily through contaminated water and soil [7] and PI3 is primarily transmitted by aerosolization [20]. We found that site explained exposure to PI3 potentially because densities of deer and cattle are higher in the Eastern study area, and PI3 is transmitted from animal to animal. At this time, we do not have information regarding water sources or contamination associated with stagnant water on public land, so we can only speculate as to the cause of the relationships we identified for *L*. *i*. *pomona* seroprevalence. Future research focusing on potential risk factors for exposure to *L*. *i*. *pomona* is warranted, including contaminated water sources and contact with cattle.

## Supporting Information

S1 File Raw data used for models explaining exposure of white-tailed deer in Wisconsin to parainfluenza 3 and *Leptospira interrogans pomona*. (XLSX)
